# Genome sequencing unveils mutational landscape of the familial Mediterranean fever: Potential implications of IL33/ST2 signalling

**DOI:** 10.1111/jcmm.15701

**Published:** 2020-08-27

**Authors:** Meenakshi Umar, Andre Megarbane, Jingxuan Shan, Najeeb Syed, Eliane Chouery, Elbay Aliyev, Puthen Jithesh, Ramzi Temanni, Issam Mansour, Lotfi Chouchane, Aouatef Ismail Chouchane

**Affiliations:** ^1^ Laboratory of Inflammation Research Immunology Department Sidra Medicine Doha Qatar; ^2^ Institut Jérôme Lejeune Paris France; ^3^ Centre Medical et Psychopedagogique Beirut Lebanon; ^4^ Laboratory of Genetic Medicine and Immunology Weill Cornell Medicine‐Qatar Qatar; ^5^ Department of Genetic Medicine Weill Cornell Medicine New York NY USA; ^6^ Biomedical Informatics Division Sidra Medicine Doha Qatar; ^7^ Medical School Lebanese American University Beirut Lebanon; ^8^ Human Genetics Department Sidra Medicine Doha Qatar; ^9^ American University of Science and Technology (AUST) Beirut Lebanon; ^10^ Department of Microbiology & Immunology Weill Cornell Medicine New York NY USA

**Keywords:** Familial Mediterranean Fever, *IL1RL1*, *MEFV*, Whole Genome Sequencing

## Abstract

Familial Mediterranean fever (FMF) is the most common auto‐inflammatory disease. It is transmitted as autosomal recessive trait with mutations in MEditerranean FeVer (*MEFV)* gene. Despite a typical clinical expression, many patients have either a single or no mutation in *MEFV*. The current work is aimed to revisit the genetic landscape of FMF disease using high‐coverage whole genome sequencing. In atypical patients (carrying a single or no mutation in *MEFV*), we revealed many rare variants in genes associated with auto‐inflammatory disorders, and more interestingly, we discovered a novel variant ( a 2.1‐Kb deletion) in exon 11 of *IL1RL1* gene, present only in patients. To validate and screen this patient‐specific variant, a tandem of allele‐specific PCR and quantitative real‐time PCR was performed in 184 FMF patients and 218 healthy controls and we demonstrated that the novel deletion was absent in controls and was present in more than 19% of patients. This study sheds more light on the mutational landscape of FMF. Our discovery of a disease‐specific variant in *IL1RL1* gene may constitute a novel genetic marker for FMF. This finding suggesting a potential role of the IL33/ST2 signalling in the disease pathogenicity highlights a new paradigm in FMF pathophysiology.

## 1 INTRODUCTION

Auto‐inflammatory diseases (AIDs) are a distinct group of disorders characterized by an unprovoked systemic inflammation without the presence of high titre of autoantibodies nor antigen‐specific T cells.^1^, ^2^ Most of the AIDs are monogenic and are caused by highly penetrant mutations in single genes encoding proteins involved in the innate immunity, but complex and polygenic AIDs with significant environmental influence have also been identified.^3^

Familial Mediterranean fever (FMF) is the most common Mendelian auto‐inflammatory disease, characterized by uncontrolled activation of the innate immune system, resulting in recurrent brief episodes of fever and serositis with chest, abdominal, joints and muscles pain.^4^ Predominantly, FMF affects people from Mediterranean and Middle Eastern ethnic origins (1/200‐1/1000).^5^

The causing gene of FMF is the MEditerranean FeVer (*MEFV*) gene.^5^, ^6^ The *MEFV* gene encodes 781 amino acids pyrin (or marenostrin) protein, which is mostly expressed in neutrophils, eosinophils, monocytes, dendritic cells and fibroblasts.^7^, ^8^ The exact physiological role of pyrin protein is not clear; however, it is suggested to play a role in apoptosis, inflammation, cytokine production and innate immune response. The *MEFV* gene, located on chromosome 16p13.3, is approximately 14.6 kb long and contains 10 exons. The gene can harbour multiple mutations in different exons; however, exon 2 and exon 10 are two mutational hot spots, with exon 10 having the largest number of mutations. The five founder mutations are p.Met694Val, p.Met694Ile, p.Val726Ala and p.Met680Ile present in exon 10 and p.Glu148Gln in exon 2, together they represent more than 80% of the disease‐causing mutations.^9^

The analysis of the typical FMF patients revealed an autosomal recessive model of inheritance.^5^ The disease can segregate either in homozygous or in a compound heterozygous modality. However, it is observed that a substantial number of FMF patients are either heterozygous or carry no *MEFV* mutation. The possibility of pseudo‐dominance is considered in rare cases but it is yet to be proven and it could not explain the large number of clinical FMF cases.^10^, ^11^ The hypothesis of digenic or oligogenic inheritance is gaining attention and could explain the divergence of clinical FMF with single or no mutation in *MEFV* gene from the typical paradigm of recessive inheritance.^12^, ^13^ The presence of mutations in modifier genes associated with inflammation or interactions between *MEFV* mutation and modifying allele in genes involved in known auto‐inflammatory diseases, as reported in a limited number of studies, could also be responsible for the large spectrum of FMF phenotypes.^14^, ^15^

The lack of comprehensive genetic analyses of FMF patients with single or no mutated allele in *MEFV* gene is prompting us to investigate the genetic landscape of FMF disease in a large cohort of FMF patients with different *MEFV* mutational profiles using both Sanger and whole genome sequencing (WGS).

## 2 METHODS

### 2.1 Patients and controls

The study population consisted of 402 unrelated Lebanese subjects including 184 FMF patients (102 males and 82 females with median age 17 ± 5 years) recruited from several medical centres in Beirut, Lebanon, and 218 gender and ethnicity matched healthy controls recruited among subjects visiting the hospitals for routine health check‐up and who were free from any chronic inflammatory and autoimmune disease. Blood sample collection and storage was managed by the Medical Center CEMEDIPP and the American University of Science and Technology in Beirut, Lebanon. The diagnosis of FMF in our patients was made according to the established criteria of both Sohar (Tel Hashomer criteria) ^5^ and Livneh.^16^ More stringent clinical diagnosis criteria were used to establish the diagnosis of FMF in patients with a single disease‐causing *MEFV* variant or with no identified *MEFV* variants. The 184 FMF patients were randomly selected from a large cohort of patients for whom Sanger sequencing of 10 exons of *MEFV* gene was performed, and who, based on copies of *MEFV* mutated allele, were stratified into three groups: (a) zero mutation: patients without any mutation in *MEFV* gene; (b) single mutation: patients with only one mutation in *MEFV* gene; and (c) double mutation: patients with two *MEFV* mutations. In order to increase the chance to identify novel and/or modifier genes for FMF, we purposely enriched our study cohort with more patients with a single or no variant in *MEFV* gene. We performed WGS on 50 patient samples (11 patients with double *MEFV* mutation, 19 patients with a single mutation and 20 patients with no *MEFV* mutation) randomly selected from the 3—Sanger sequencing—defined subcategories and that of 26 healthy control subjects.

The study protocol was approved by ethics committee of Sidra Medicine, Doha, Qatar (Protocol number # 1511002018). All study subjects signed a written informed consent prior to be enrolled in the study.

### 2.2 Sample preparation and whole genome sequencing (WGS)

Peripheral blood samples were collected from patients and controls in EDTA tubes and genomic DNA was extracted by standard salt‐precipitation methods.^17^ WGS was carried on DNA of 50 FMF cases along with 26 controls with a HiSeq 2500 sequencer (30× average coverage) at Sidra Medicine, Qatar. Paired‐end libraries were generated from 1 μg of genomic DNA using an Illumina TruSeq DNA PCR‐Free Sample Preparation Kit. Genomic DNA was sheared using a Covaris system. Isolated DNA fragment ends were blunted, A‐tailed and ligated with sequencing adaptors with index sequences. Excess adapters and enzymes were removed using AMPure beads (Beckman Coulter Genomics). Indexed libraries were size‐selected to the 350 bp range using bead‐based capture, and the concentration of amplifiable fragments was determined by qPCR, relative to sequencing libraries with a known concentration. Normalized libraries were clustered on a c‐BOT machine, and 125 bp paired‐end sequencing was performed on the HiSeq 2500 system.

### 2.3 WGS data analysis

Paired‐end raw fastq files were mapped to the reference human genome, build GrCh37, using BWA‐MEM aligner: 0.7.12‐r1039,^18^ GATK Haplotype caller was used for variant calling on individual samples. GATK Genotype GVCFs option was used for joint calling across individual samples. Variant calling was performed using recommended best practices of GATK version 3.7. Joint variant file was further gone through with GATK variant quality score recalibration (VQSR) step.^19^ The annotation of variants was performed by using SNPEFF (version: 4.3r, GRCh37.75 Reference Build) and dbNFSP 3.0.^20^ Ingenuity® Variant Analysis ([https://www.qiagenbioinformatics.com/products/ingenuity‐variant‐analysis)] from QIAGEN, Inc”) was used to filter variants based on various parameter: (a) Variants with low‐call quality (<20), low coverage (<10), which failed in VQSR filter and which were present in low complexity region were excluded, (b) variants with allele frequencies more than 1% in public database including 1000G phase3,^21^ gnomAD version 2.1.1 ^22^ and ExAc project release 1 ^23^ were excluded unless established as a pathogenic variant, (c) homozygous, heterozygous or compound heterozygous variants which were present in cases and absent in controls were selected and (d) only non‐synonymous, frameshift, non‐sense and splice site variants, which could be potential deleterious based on CADD version 1.3 score (>20) and functional predictions by SIFT version 5.1.1 and Polyphen‐2 version 2.2r398, were selected.^24^, ^25^, ^26^ Furthermore, variants, which were either related to auto‐inflammatory diseases including FMF or which were reported to interact with known genes associated with auto‐inflammatory diseases, were chosen.

For copy number variant (CNV) analysis, we used three structural variant callers: Delly version 0.7.8, Speedseq version 0.1.2 and GenomeSTRiP version 2.00.171, and we applied the best practices recommended by authors of the tools. The annotation of structural variant was carried out using AnnTools version 1.0.^27^ Only rare, exonic structural variants, which were absent in controls, were selected for further analysis. For the visualization and confirmation of structural variants, we used SAMPlot (https://github.com/ryanlayer/samplot).

We have submitted all the variants reported here to LOVD website (https://www.lovd.nl).

### 2.4 Genetic screening for the novel variant of *IL1RL1* gene

Screening for the presence of the novel variant (2.1‐Kb deletion) of *IL1RL1* gene (NM_016232, NC_000002.11:g.102967165_102969288del), identified by WGS, was performed in all 402 subjects using a tandem of 2 PCR assays (allele‐specific PCR [AS‐PCR] followed by a quantitative real‐time PCR (qRT‐PCR)]). First, samples are analysed by AS‐PCR using primers flanking a genomic region of 3 Kb encompassing the 2.1‐Kb deletion. A simultaneous amplification of a 3‐Kb fragment and a 0.9‐Kb fragment corresponds to the presence of a heterozygous deletion of exon 11 of the *IL1RL1* gene, and an amplification of a 3‐Kb fragment only indicates the absence of such deletion. In order to confirm the outcome of the AS‐PCR, a qRT‐PCR was performed to quantify the copy number of the region flanking the 2.1‐Kb deletion.

Briefly, 50 ng of genomic DNA was subjected to a total of 25 μL PCR containing 200μM dNTP, 0.5 μmol/L each of forward and reverse primer and 0.5 unit Phusion® High‐Fidelity DNA polymerase (NEB), with a PCR program of 95°C for 1’30’’, followed by 35 cycles at 94°C for 25”, 65°C for 30” and 72” for 1’40” in a Veriti Thermal Cycler (Applied Biosystems). Primers were designed to amplify a 3.0‐Kb fragment encompassing the 2.1‐Kb deletion: Forward primer 5’‐ TCTCACACTCAAGCTTGTGCTG‐3’ and reverse primer 5’‐AGAGCTCTCATACACAACTGGTG‐3’. All PCR products were examined by electrophoresis on 1.5% agarose gels and photographed with a ChemiDoc^TM^ MP Imaging System (Bio‐Rad).

To confirm the outcome of the AS‐PCR, the qRT‐PCR was performed using two sets of pair of primers; one set was used to amplify a DNA fragment within the 2.1‐Kb deletion (forward primer 5’‐AGAAGCAATAGTGCCTGCTG‐3’ and reverse primer 5’‐ATTCCTGCTCCTCACACTTC‐3’), and another set to amplify, as an endogenous control, a DNA fragment upstream the 2.1‐Kb deletion (forward primer 5’‐AACGGCTCAAGAGACTTGTG‐3’ and reverse primer 5’‐TACTTCTACCTGCATGGGTG‐3’). The qRT‐PCR was performed in a total volume of 20 μL containing 15 ng genomic DNA, 10μl GoTaq® qPCR Master Mix (Promega) and 0.5 μmol/L each of forward and reverse primer using a cycling program of 2’ at 50°C, 2’ at 95°C, 40 cycles consisting of 15” at 95°C and 45” at 60°C, and a dissociation curve analysis step of 15” of a rapid ramp to 95°C, 15” at 60°C and 15” of a slow ramp to 95°C on a QuantStudio 6 Flex Real‐Time PCR system (Applied Biosystems) in Fast 96‐well plate format. qPCR for each amplicon of each patient was performed in triplicate, and AS‐PCR–verified WGS patients with and without the 2.1‐Kb deletion were included for each plate as controls. The results were analysed using the comparative C_T_ (ΔΔC_T_) method.

Chi‐square test was used to compare the frequency between the two groups of patients (patients with a single or no *MEFV* mutation *vs* patients with 2 *MEFV* mutations), and the Phi coefficient was used to generate the effect size of this novel variant in patients.

## 3 RESULTS

### 3.1 Characterization of *MEFV* mutations in patients with FMF

The 184 FMF patients of the present study were randomly selected from a large cohort for which Sanger sequencing of coding sequence of *MEFV* gene was performed. In order to increase the chance to unveil novel pathogenic and/or modifiers genes for FMF, we purposely enriched the patient population with more patients carrying single or no mutation in *MEFV* gene. Out of the 184 FMF cases, 58 (31.5%) patients had biallelic variants of the *MEFV* gene, 57 (31.0%) patients were heterozygous, while 69 (37.5%) patients did not carry any coding mutations in *MEFV* gene. The mutational analysis showed that the Met694Val mutation was the most frequent mutation, followed by the Val726Ala, p.Pro158Ser/p.Pro369Ser, p.Arg197Gln/p.Arg408Gln and Met694Ile. This result is in agreement with previous studies.^28^, ^29^

### 3.2 WGS and the search of novel pathogenic or modifier genes for FMF

To investigate the potential presence of variants in novel pathogenic and/or modifiers genes in FMF patients with single or no mutated allele in *MEFV* gene, we analysed the WGS data of 50 patients, randomly selected from the 3 subcategories of patients, and that of 26 healthy control subjects.

**FIGURE 1:**
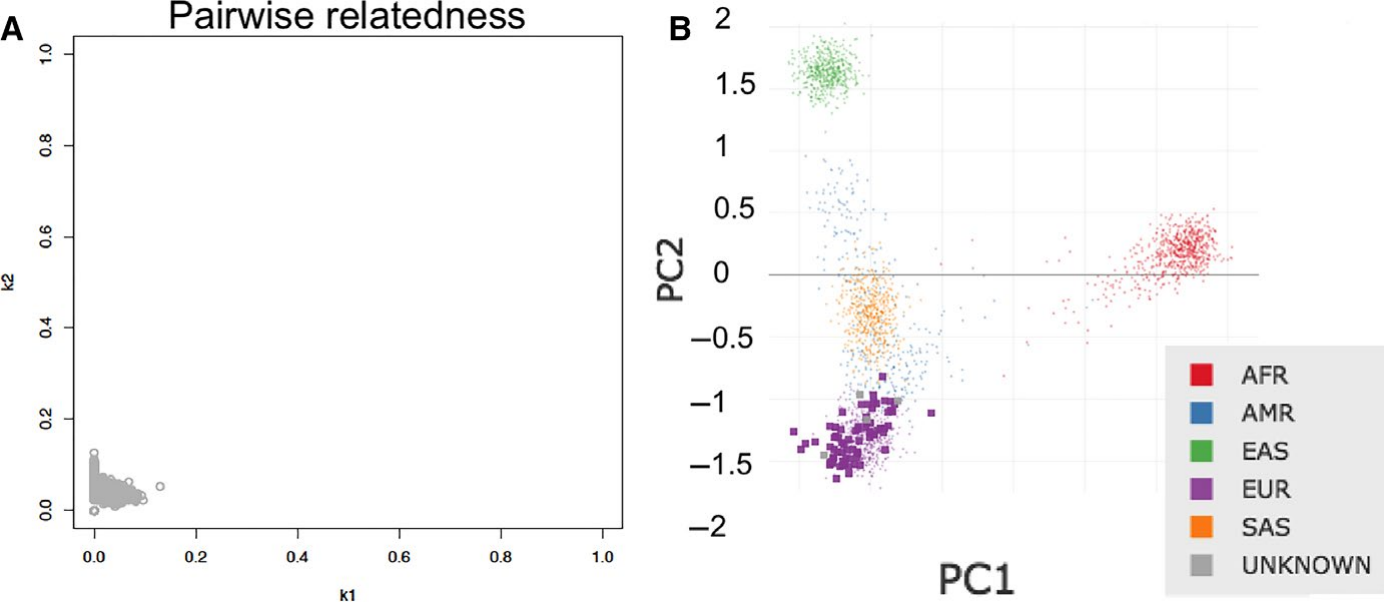
(A) Identity by descent (IBD) plot displaying un‐relatedness of the 76 samples on which whole genome sequencing was performed. (B) Principal component analysis (PCA) plot of the same samples mapped on 1000‐Genome data set; AFR = African, AMR = Ad Mixed American, EAS = East Asian, EUR = European, SAS = South Asian

We performed the identity by descent (IBD) estimation ^30^ in the 76 samples, which indicated that our study subjects were unrelated (Figure 1A). Principal component analysis ^31^ was performed on the 76 samples along with samples from the 1000 Genomes Project data set, revealing a genetic signature with proximity to that of the European ancestry (Figure 1B).

The status of *MEFV* mutations in patients, initially defined by Sanger sequencing, was confirmed by WGS. The list of all *MEFV* variants, identified by WGS, found in the 50 patients with FMF is shown in Table 1. The *MEFV* variants were exclusively present in FMF cases and were absent in controls. In addition, WGS revealed in our patients three novel variants in the promoter region of *MEFV* gene: c.‐123A > G, c.‐397C > G and c.‐1309G > A (reference sequence: NC_000016.9). These heterozygous promoter variants were present in only 3 FMF cases and were predicted to cause loss of the promoter function of the gene. Beside non‐synonymous and promoter variants, two synonymous heterozygous variants (p.Pro124Pro and p.Arg290Arg/p.Arg501Arg) were found in FMF cases.

**TABLE 1:** List of the *MEFV* variants revealed by WGS and present in patients with FMF

Position at Chr 16	Gene Region	Mutation description	Cases (N)	Genotype (Patient ID)	Regulatory site	Regulator	Frequency in gnomAD
3293257	Exon 10	NM_000243.2:c.2230G > T (p.Ala744Ser)	2	Het (FMF16 & 30)			0.0018
3293310	Exon 10	NM_000243.2:c.2177T > C (p.Val726Ala)	10	Het (FMF1, 5, 6,7,11, 27, 28); Homo (FMF 3, 4, 10);			0.0020
3293403	Exon 10	NM_000243.2:c.2084A > G (p.Lys695Arg)	2	Het (FMF17, 26)			0.0058
3293405	Exon 10	NM_000243.2:c.2082G > A (p. Met694Ile)	3	Het (FMF8, 14, 21)			0.0001
3293407	Exon 10	NM_000243.2:c.2080A > G (p.Met694Val)	12	Homo (FMF2); Het (FMF1, 6, 7, 12, 13, 15, 18, 19, 23, 24, 25)			0.0003
3293447	Exon 10	NM_000243.2:c.2040G > A (p.Met680Ile)	2	Het (FMF8, 11)			0.0 000 079
3299468^a^	Exon3	NM_000243.2:c.1223G > A (p.Arg408Gln)	4	Het (FMF9, 20, 22, 29)			0.0134
3299586^a^	Exon3	NM_000243.2:c.1105C > T (p.Pro369Ser)	4	Het (FMF9, 20, 22, 29)			0.0147
3304 626	Exon2	NM_000243.2:c.442G > C (p.Glu148Gln)	2	Het (FMF5, 9)			0.0658
3306710^b^	Promoter	NC_000016.9:g.3306710T > C (NG_007871.1:g.4918A > G (c.‐123A > G))	1	Het (FMF31)	Encode TFBS, Promoter Loss	CEBPB, HNF1B, REST, STAT3	NA
3306984^b^	Promoter	NC_000016.9:g.3306984G > C (NG_007871.1:g.4644C > G (c.‐397C > G))	1	Het (FMF12)	Promoter Loss	HNF1B	NA
3307896^b^	Promoter	NC_000016.9:g.3307896C > T (NG_007871.1:g.3732G > A (c.‐1309G > A))	1	Het (FMF13)	Promoter Loss	NFIC	NA

Abbreviations: Chr, chromosome; gnomAD, Genome Aggregation Database version 2.1.1; Het, Heterozygous variant; Homo, Homozygous variant; NA: Not available.

^a^ Arg408Gln variant is in cis with Pro369Ser.

^b^ Indicates novel variant.

### 3.3 Mutational Spectrum of genes associated with other AIDs in FMF patients

After filtering out variants which had high prevalence in the general population (allele frequency > 1%) or were present in controls, we first examined variants in known AID‐associated genes. More than 50 genes associated with auto‐inflammatory disorders were selected from Systemic autoinflammatory disease (SAID; http://www.autoinflammatory‐search.org/)) and Infever database.^32^ The list of the novel variants of genes associated with AIDs found in the 50 FMF patients is shown in Table 2. We observed that 10 out of the 50 FMF cases had variants in genes linked with familial haemophagocytic lymphohistiocytosis (FHL), with *PRF1* NM_005041.4:c.272C > T (p.Ala91Val), NM_005041.4:c.1153C > T (p.Arg385Trp) and *STXBP2* NM_006949.3:c.1034C > T (p.Thr345Met) variants present in 3 patients each, while variants in other known FHL‐associated genes (*RAB27A* and *UNC13D*) were present in one FMF case each. *PRF1* p.Ala91Val variant is classified as DFP (ie disease‐associated polymorphism with additional functional evidence) in the Human Gene Mutation Database (HGMD) ^33^ for FHL. Screening for genes associated with hereditary fever syndromes other than *MEFV* revealed also the presence of variants in *PSTPIP1* (NM_003978.4:c.203C > A (p.Thr68Lys) and a splice site variant NC_000015.9(NM_003978.4):c.37‐10081C > G), *TNFRSF11A* (NM_003839.3:c.1234G > T (p.Asp412Tyr), NM_003839.3:c.1348C > T (p.Arg450Trp)) and in *NLRP3* (NM_001079821.2:c.2861C > T (p.Thr954Met)). Furthermore, four different variants in *NOD2* gene (NM_022162.2:c.2230C > T (p.Arg744Trp), NM_022162.2:c.2127G > A (p.Trp709*), NM_022162.2:c.676_691del (p.Arg227fs*145) and NC_000016.9(NM_022162.2):c.2883‐2A > G) were also observed in FMF cases. Other auto‐inflammatory disorder genes, which had missense substitution in our present cohort of FMF cases, were *IFIH1* (NM_022168.3:c.1126G > A (p.Glu376Lys), and NM_022168.3:c.2597C > T (p.Pro866Leu)), *PLCG2* (NM_002661.4:c.82A > T (p.Met28Leu)), *TNFAIP3* (NM_001270508.1: c.406C > T (p.Arg136Cys)) and *SH3BP2* (NM_001145856.1:c.1600C > T (p.Arg534Trp)). We observed also three predicted pathogenic variants in genes associated with Psoriasis 2 and 15 (*CARD14* NM_024110.4:c.1789C > T (p.Arg597Trp) and NM_024110.4:c.1789C > T (p.Arg597Trp) and *AP1S3* NM_001039569.1:c.11T > G (p.Phe4Cys)) in FMF cases, with *AP1S3* (p.Phe4Cys) listed as disease‐causing mutation (DM) for psoriasis 15 in the HGMD.

**TABLE 2:** List of variants of auto‐inflammatory disorders genes found in FMF patients

Gene	Variant details	Chr: position	Type of Mutation	dbSNP ID	Cases (N)	SIFT	Polyphen‐2	CADD Score	Frequency in gnomAD	Acronym of SAID		AID Mode of Inheritance
*PRF1*	NM_005041.4:c.272C > T (p.Ala91Val)	10:72 360 387	Missense	rs35947132	3	Damaging	Probably Damaging	26	0.0292		FHL	Autosomal recessive
*PRF1*	NM_005041.4:c.1153C > T (p.Arg385Trp)	10:72 358 324	Missense	rs72358324	3	Damaging	Probably Damaging	20.8	0.0002		FHL	
*STXBP2*	NM_006949.3:c.1034C > T (p.Thr345Met)	19:7 708 058	Missense	rs117761837	3	Damaging	Probably Damaging	26.3	0.0106		FHL	
*RAB27A*	NM_004580.4:c.17A > G (p.Tyr6Cys)	15:55 527 116	Missense	rs145253993	1	Damaging	Probably Damaging	27	0.0001		FHL	
*UNC13D*	NM_199242.2:c.670C > T (p.His224Tyr)	17:73 836 856	Missense	rs145607492	1	Damaging	Probably Damaging	25.5	0.0002		FHL	
*UNC13D*	NM_199242.2:c.610A > G (p.Met204Val)	17:73 837 042	Missense	rs144722609	1	Tolerated	Benign	22.6	0.0007		FHL	
*TNFAIP3*	NM_001270508.1:c.406C > T (p.Arg136Cys)	6:138 196 092	Missense	rs200740561	1	Damaging	Probably Damaging	35	0.0001		AISBL	Autosomal dominant
*PSTPIP1*	NM_003978.4:c.203C > A (p.Thr68Lys)	15:77 310 863	Missense	NA	1	Damaging	Possibly Damaging	25.7	NA		PAPA	Autosomal dominant
*PSTPIP1*	NC_000015.9(NM_003978.4):c.37‐10081C > G	15:77 300 408	Splice site^a^	rs1020233393	1	NA	NA	<10	NA		PAPA	
*NOD2*	NM_022162.2:c.2230C > T (p.Arg744Trp)	16:50 746 052	Missense	rs140876663	1	Damaging	Probably Damaging	26.8	0.0001		Blau syndrome	Autosomal dominant
*NOD2*	NM_022162.2:c.2127G > A (p.Trp709*)	16:50 745 949	Stop gain	rs776701942	1	NA	NA	35	0.000008		Blau syndrome	
*NOD2*	NM_022162.2:c.679_694del (p.Arg227fs*145)	16:50 744 498	Frameshift	NA	1	NA	NA		NA		Blau syndrome	
*NOD2*	NC_000016.9(NM_022162.2):c.2883‐2A > G	16:50 759 398	Splice Site	rs564226539	1	NA	NA	24.8	0.00002		Blau syndrome	
*TNFRSF11A*	NM_003839.3:c.1234G > T (p.Asp412Tyr)	18:60 036 384	Missense	NA	1	Damaging	Probably Damaging	24.5	NA		TRAPS11	Autosomal dominant
*TNFRSF11A*	NM_003839.3:c.1348C > T (p.Arg450Trp)	18:60 036 498	Missense	rs34945627	1	Damaging	Probably Damaging	22.9	0.0009		TRAPS11	
*NLRP3*	NM_001079821.2:c.2861C > T (p.Thr954Met)	1:247 607 973	Missense	rs139814109	1	Damaging	Probably Damaging	33	0.0012		CAPS	Autosomal dominant.
*IFIH1*	NM_022168.3:c.1126G > A (p.Glu376Lys)	2:163 139 056	Missense		1	Damaging	Probably Damaging	33	NA		AGS7	Autosomal recessive
*IFIH1*	NM_022168.3:c.2597C > T (p.Pro866Leu)	2:163 128 755	Missense	rs200833729	1	Tolerated	Possibly Damaging	23.0	0.0004		AGS7	
*PLCG2*	NM_002661.4:c.82A > T (p.Met28Leu)	16:81 819 676	Missense	rs61749044	1	Tolerated	Possibly Damaging	24.0	0.0106		APLAID	Autosomal dominant
*SH3BP2*	NM_001145856.1:c.1600C > T (p.Arg534Trp)	4:2 834 080	Missense	rs14876133	2	Damaging	Probably Damaging	32	0.0043		Cherubism	Autosomal dominant
*CARD14*	NM_024110.4:c.1789C > T (p.Arg597Trp)	17:78 172 328	Missense	NA	1	Damaging	Probably Damaging	34	0.0037		PSORS2	Autosomal dominant
*CARD14*	NM_024110.4:c.239G > A (p.Arg80Gln)	17:78 156 479	Missense	NA	1	Tolerated	Probably Damaging	25	NA		PSORS2	
*AP1S3*	NM_001039569.1:c.11T > G (p.Phe4Cys)	2:224 642 579	Missense	rs116107386	1	Damaging	Probably Damaging	27.2	0.0079		PSOR15	Autosomal dominant

Abbreviations: AGS7, Aicardi‐Goutieres syndrome 7; AIBSL, autoinflammatory syndrome, familial, Behcet‐like; APLAID, auto‐inflammation and PLCG2‐associated antibody deficiency and immune dysregulation; CAPS, cryopyrin‐associated periodic syndromes; Chr, chromosome; FHL, familial haemophagocytic lymphohistiocytosis; gnomAD, Genome Aggregation Database version 2.1; NA, not available; PAPA, pyogenic sterile arthritis, pyoderma gangrenosum and acne syndrome; PSOR15, pustular psoriasis; PSOR2, familial psoriasis; TRAPS11, TNFRSF11A‐associated hereditary fever disease.

All the listed variants were present in heterozygous state in FMF cases and were absent in controls; software version: SIFT version 5.1.1, PolyPhen‐2 version 2.2.2r398, CADD version 1.3.

^a^ Results in splice site Loss.

### 3.4 Identification of novel variants in inflammatory genes in FMF patients

Variants in known AID‐associated genes identified in our cohort were not sufficient to completely draw the genetic variation pattern in our FMF patients. We further looked for the predicted pathogenic variants in inflammatory genes either interacting with known genes associated to AIDs or involved in auto‐inflammation processes, using knowledge base of Ingenuity variant analysis. The list of variants in inflammatory genes found in the 50 FMF patients is shown in Table 3. We observed that *IFNAR2* (NM_207585.2:c.611C > G: (p.Thr204Arg)) was the most common variant among FMF cases, and it was present in 7 out of 50 FMF cases (from the three FMF subgroups). IFNAR2 associates with IFNAR1 to form a receptor for interferons alpha (*IFNA1*) and beta (*IFNB1*). In the present study, FMF cases also had variants in *IFNAR1* (NM_000629.2:c.954G > C (p.Trp318Cys)) and in *IFNB1* (NM_002176.3:c.498A > G (p.Ile166Met)), which were present in two FMF cases and one FMF case, respectively. A more comprehensive screening from the list of inflammatory genes identified from Ingenuity revealed that our FMF patients had many variants in genes of the superfamily of TNF and its receptors. A stop gain variant in *TNFRSF4* (NM_003327.3:c.384C > A (p.Cys128*)) was present in two FMF cases, whereas missense variants in *TNFRSF8* (NM_001243.4:c.1511G > A (p.Arg504Gln)) and *TNFRSF9* (NM_003811.3:c.716G > A (p.Arg239Gln)) were present in single FMF case each. We also identified two variants in genes involved in TLR pathway: *TLR1* (NM_003263.3:c.1013T > C (p.Met338Thr)) and *TRAFD1* (NM_001143906.1:c.908A > C (p.Glu303Ala). Many interleukins and their receptors sequences were also found to be altered in FMF patients like *IL17RB* (NM_018725.3:c.529G > A (p.Gly177Arg)), *IL17RD* (NM_017563.4:c.1696C > T (p.Pro566Ser)), *IL1R2* (NM_004633.3:c.932T > C (p.Ile311Thr)), *IL20* (NC_000001.10(NM_018724.3):c.225 + 1G>T), *IL12A* (NM_000882.3:c.631G > A (p.Val211Met)) and *IL1A* (NM_000575.4:c.526G > C (p.Asp176His)), with *IL17RB* (NM_018725.3:c.529G > A (p.Gly177Arg)) and *IL1R2 (*NM_004633.3:c.932T > *C* (p.Ile311Thr)) variants present in three patients each, and the remaining other variants present in one case each. Among NLR family of genes, *NLRC3* NM_178844.3:c.2401G > A: (p.Ala801Thr), *NLRP2* NM_017852.4:c.2672G > T (p.Gly891Val) and *NLRX1* NM_024618.3:c.1480G > A (p.Val494Met) were present in one FMF case each. A missense variant in *CASP14* gene (NM_012114.2:c.418G > A (p.Gly140Ser)) was observed in one FMF case. Endoplasmic reticulum aminopeptidases genes, *ERAP1* and *ERAP2*, which encode proteins involved in peptide trimming for HLA class I molecules,^34^ were altered in four and one FMF cases, respectively. Some other predicted pathogenic variants in FMF cases were *LILRB1* NM_006669.6:c.997G > T (p.Gly333Cys), *RAB27B* NM_004163.4:c.274G > A (p.Ala92Thr) and *ICAM1* NM_000201.2:c.1099C > T (p.Arg367Cys).

**TABLE 3:** List of variants in inflammatory genes in FMF patients

Gene	Variant details	Chr: position	Type of variant	dbSNP ID	Cases (N)	SIFT	Polyphen‐2	CADD Score	Frequency in gnomAD
*IFNAR2*	NM_207585.2:c.611C > G (p.Thr204Arg)	21:34 625 037	Missense	rs147496374	7	Damaging	Probably Damaging	29.3	0.0046
*IFNAR1*	NM_000629.2:c.954G > C (p.Trp318Cys)	21:34 721 562	Missense	rs578193831	2	Damaging	Probably Damaging	28.7	0.00 002
*IFNB1*	NM_002176.3:c.498A > G (p.Ile166Met)	9:21 077 371	Missense	rs141894933	1	Damaging	Probably Damaging	15.94	0.0016
*TNFRSF4*	NM_003327.3:c.384C > A (p.Cys128^a^)	1:1 148 071	Stop gain	NA	2	NA	NA	36	NA
*TNFRSF8*	NM_001243.4:c.1511G > A (p.Arg504Gln)	1:12 198 461	Missense	rs2230627	1	Tolerated	Probably Damaging	28.8	0.0002
*TNFSF9*	NM_003811.3:c.716G > A (p.Arg239Gln)	19:6 535 028	Missense	rs755292822	1	Tolerated	Probably Damaging	23.3	NA
*TRAFD1*	NM_001143906.1:c.908A > C (p.Glu303Ala)	12:112 583 447	Missense	rs79680080	1	Damaging	Probably Damaging	26	0.0158
*TLR1*	NM_003263.3:c.1013T > C (p.Met338Thr)	4:38 799 440	Missense	rs990267834	1	Damaging	Possibly Damaging	23.5	NA
*IL1R2*	NM_004633.3:c.932T > C (p.Ile311Thr)	2:102 642 617	Missense	rs144482163	3	Damaging	Probably Damaging	26.1	0.0023
*IL1A*	NM_000575.4:c.526G > C p.(Asp176His)	2:113 535 653	Missense	rs1801715	1	Damaging	Probably Damaging	23.3	0.00 006
*IL12A*	NM_000882.3:c.631G > A (p.Val211Met)	3:159 713 215	Missense	rs35990253	1	Tolerated	Probably Damaging	18.74	0.0040
*IL17RB*	NM_018725.3:c.529G > A (p.Gly177Arg)	3:53 889 368	Missense	rs2232337	3	Damaging	Probably Damaging	29.7	0.0044
*IL17RD*	NM_017563.4:c.1696C > T (p.Pro566Ser)	3:57 132 035	Missense	rs61742267	1	Tolerated	Probably Damaging	23.6	0.0142
*IL20*	NC_000001.10(NM_018724.3):c.225 + 1G>T	1:207 039 710	Splice site^a^	rs138566326	1	NA	NA	23.5	0.0006
*NLRP2*	NM_017852.4:c.2672G > T (p.Gly891Val)	19:55 502 004	Missense	NA	1	Tolerated	Probably Damaging	22.1	NA
*NLRC3*	NM_178844.3:c.2401G > A: (p.Ala801Thr)	16:3 600 448	Missense	rs767176921	1	Damaging	Possibly Damaging	24.4	0.00 001
*NLRX1*	NM_024618.3:c.1480G > A (p.Val494Met)	11:119 045 792	Missense	rs780397677	1	Tolerated	Probably Damaging	23.7	0.0000
*CASP14*	NM_012114.2:c.418G > A (p.Gly140Ser)	19:15 165 983	Missense	rs761542772	1	Damaging	Probably Damaging	25.2	0.00 004
*LILRB1*	NM_006669.6:c.997G > T (p.Gly333Cys)	19:55 144 505	Missense	rs201421803	1	Damaging	Probably Damaging	22.7	0.0006
*RAB27B*	NM_004163.4:c.274G > A (p.Ala92Thr)	18:52 551 598	Missense	rs9962265	1	Damaging	Probably Damaging	34.0	0.000 003
*ICAM1*	NM_000201.2:c.1099C > T (p.Arg367Cys)	19:10 395 252	Missense	rs139178890	1	Damaging	Probably Damaging	32	0.0005
*ERAP2*	NM_022350.4:c.1040C > T (p.Thr347Met)	5:96 228 072	Missense	rs75263594	4	Damaging	Probably Damaging	26.8	0.02 155
*ERAP1*	NM_016442.4:c.1378G > C (p.Gly460Arg)	5:96 126 289	Missense	rs771994807	1	Damaging	Probably Damaging	27.8	0.000 015

Abbreviations: Chr, Chromosome; NA, Not available.

Note: All the listed variants were present in heterozygous state in FMF cases and were absent in controls.

Software version: SIFT version 5.1.1, PolyPhen‐2 version 2.2.2r398, CADD version 1.3.

^a^ Splice site loss

### 3.5 Copy number variant (CNV) analysis in FMF

Beside point mutations and small indels, we also looked for the structural variants in the whole genome of the 50 FMF cases. Variant calling was done using Delly version 0.7.8, GenomeSTRiP version 2.00.17.1 and Speedseq version 0.1.2 using best practices recommended by authors of the tools. Later, final output from these 3 tools annotated with Anntools version 1.0. After removing variants, which either were present in controls or were located in non‐coding regions, 164 deletions were identified by GenomeSTRiP version 2.00.17.1, 704 variants (358 deletions, 334 duplications, 12 inversions) were found by Speedseq version 0.1.2 and 1178 variants (338 duplications, 398 deletions, 442 inversions) were identified by Delly version 0.7.8. For genotyping structural variants, we used their respective genotyper modules or tools such as SVTyper for speedseq, SVGenotyper module of GenomeSTRiP and integrated genotyper of Delly. We performed manual inspection of all these variants and found a deletion in *IL1RL1* gene, which was consistently detected by all three software. This heterozygous deletion in exon 11 of *IL1RL1* gene (NM_016232, NC_000002.11:g. 102967165_102969288 del) was around 2.1 Kb in size and was present in 9 FMF cases carrying one mutated allele of the *MEFV* gene and reported by three software on same subjects. For the visualization and confirmation of structural variants, we used SAMPlot. The representative figure of *IL1RL1* deletion in FMF cases and controls is shown in Figure S1. The search of the identified *IL1RL1* variant in the 1000G phase 3 data set showed the presence of a larger deletion of 3.1 Kb (Variant: esv3591789; http://dgv.tcag.ca/dgv/app/variant?id=esv3591789&ref=hg19), overlapping with the 2.1‐Kb *IL1RL1* deletion, in only one subject among 2504.

Table S1 shows the summary of all the variants from WGS (listed in Tables 1, 2, 3 including *IL1RL1* deletion variant) per patient to demonstrate the genotype of all FMF patients at these loci. There is no distinct pattern of distribution of AID‐associated variants and inflammatory gene variants among three subgroup of FMF patients (with zero, single and double MEFV variants) *IL1RL1* deletion variant was particularly enriched in FMF patient with single *MEFV* variant in WGS cohort. Few FMF patients had burden of several rare variants of AID and/or inflammatory genes.

### 3.6 The *IL1RL1* gene deletion in familial Mediterranean fever patients

**FIGURE 2:**
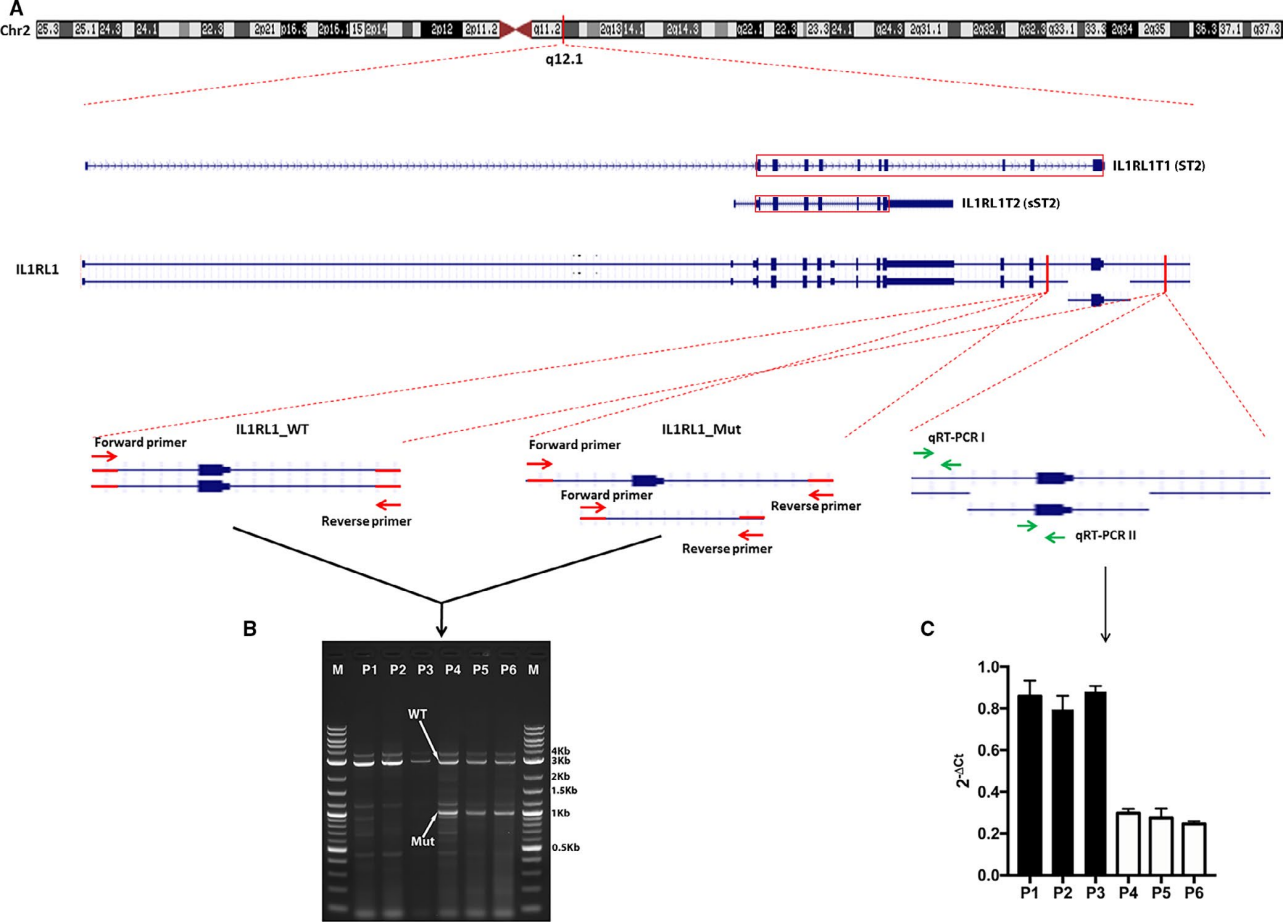
Screening of the 2.1‐Kb deletion of the *IL1RL1* gene using AS‐PCR and qRT‐PCR. (A) Schematic representation of IL1RL1 transcripts that encode ST2 and sST2, respectively, and of the *IL1RL1* heterozygous deletion containing exon 11 and experimental design to confirm the presence of the deletion using AS‐PCR and qRT‐PCR. The red box represents the coding sequence of the transcript. Hom: homozygous; Het: Heterozygous; WT: wild type; Mut: mutant. (B) DNA gel of AS‐PCR products of 6 FMF patients carrying (P4, P5 and P6) or not (P1, P2 and P3) the 2.1‐Kb deletion of exon 11 of the *IL1RL1* gene. A simultaneous amplification of a 3 Kb fragment and a 0.9 Kb fragment corresponds to the presence of the heterozygous deletion, and an amplification of a 3 Kb fragment only indicates the absence of such deletion. M: 1Kb plus DNA marker (New England Biolabs, US). (C) qRT‐PCR results of the *IL1RL1* deletion region containing exon 11 compared to its 5’ wild‐type region among 6 FMF patients. ΔCt = Ct(RT‐PCR II)‐Ct(RT‐PCR I)

To validate the finding revealed by WGS and CNV analysis, a search of the 2.1‐Kb deletion detected in the *IL1RL1* gene was performed in all 402 study subjects using allele‐specific PCR (AS‐PCR) followed by quantitative real‐time PCR (qRT‐PCR). No discrepancies in *IL1RL1* variant genotyping were found between AS‐PCR and qRT‐PCR. Figure 2 shows both the gel electrophoresis of the AS‐PCR products of samples with or without the 2.1‐Kb deletion of the *IL1RL1* gene and the quantification by qRT‐PCR of the copy number of the region flanking the 2.1‐Kb deletion. The distribution of the *IL1RL1* deletion in FMF according to the number of the mutated *MEFV* alleles is shown in Table 4. This novel variant in *IL1RL1* was found in FMF patients only. More than 19% of FMF patients are carriers of the *IL1RL1* deletion. The frequency of *IL1RL1* variant was found higher in patients with a single or no mutation in *MEFV* gene compared to that in patients carrying 2 *MEFV* mutations (0.222 vs 0.120, *P* = .05) with an effect size of 0.12. No control subject was found to be a carrier of this variant.

**TABLE 4:** Distribution of the novel variant (2.1‐Kb deletion) of *IL1RL1 gene* (NC_000002.11:g. 102967165_102969288 del) in FMF patients and in controls

Subjects (N = 402)	*IL1RL1* deletion
**Total FMF** N = 184	35 (19.02%)
***MEFV***	
2 mutations N = 58	7 (12.06%)
1 mutation N = 57	12 (21.05%)
0 mutation N = 69	16 (23.18%)
**Controls** N = 218	0 (0%)

## 4 DISCUSSION

In the present report, we showed significant genetic heterogeneity in FMF patients having single or no mutated allele of *MEFV* gene, with several patients carrying a burden of rare variants in auto‐inflammatory genes.

We first performed Sanger sequencing of coding region of *MEFV* gene in FMF cases to characterize *MEFV* mutations and to stratify patients based on the number of mutated alleles of *MEFV*. The most common *MEFV* mutation in our patient group was pMet694Val followed by p.Val726Ala, which is similar to other published reports in Lebanese and Middle Eastern populations.^28^, ^29^

As some recent familial and non‐familial studies on FMF have identified the role of selected auto‐inflammatory genes like *NLRP3*, *TNFRSF1A* and *MVK*,^35^, ^36^ we decided to screen our patients for the possibility of having rare/pathogenic mutations in other known auto‐inflammatory genes. Six broad categories of AID have been proposed based on the genetic defect in different component of the immune system: (a) IL‐1beta activation disorders (inflammasomopathies), (b) NF‐kB activation syndromes, (c) protein misfolding disorders, (d) complement regulatory diseases, (e) disturbances in cytokine signalling and (f) macrophage activation syndromes.^37^ We filtered our WGS data for the variants in more than 50 genes associated to AID belonging to one or another of the above‐mentioned AID categories and investigated for potential pathogenic variants common to FMF cases and absent in controls. Although no single variant in an AID‐associated gene seemed frequent in FMF cases, we found that six different variants in four known genes (*PRF1*, *STXBP2, RAB27A* and *UNC13D*) associated with familial haemophagocytic lymphohistiocytosis (FHL) were present in about 20% of our FMF patients. Genes associated with FHL are known to encode cytotoxic proteins: *PRF1* encodes perforin, which permeabilizes the target cell membrane, *UNC13D* encodes Munc13‐4 protein that causes cytolytic granule fusion with the cell membrane during degranulation, *RAB27A* encodes small Rab GTPase, which plays a role in exocytosis of cytotoxic vesicles, while STXBP2 is involved in the release of cytotoxic granules by natural killer cells.^38^ Mutations in these genes are supposed to impair their normal function and could lead to increased macrophages activation and cytokine production.^39^ Other AID‐associated gene variants were identified in our patients, but they were not frequent and were present only in one or two cases each.

We further investigated variations in novel genes, which are reported to interact with known auto‐inflammatory genes or which may have a role in auto‐inflammation process. The top candidate variant identified in this analysis was *IFNAR2* NM_207585.2:c.611C > G: (p.Thr204Arg), which was present in 14% of FMF cases from all subcategories (with 0, 1 and 2 *MEFV* mutations) and which is involved in type 1 interferon signalling.

Our initial search for rare structural variants in exonic regions performed on the 76 WGS (50 FMF cases and 26 controls) led to the discovery of a novel (2.1‐Kb deletion) variant in interleukin‐1 receptor‐like 1 (*IL1RL1*) gene. This deletion initially revealed by WGS was present in 9 FMF patients with a single mutated allele of the *MEFV* gene. The high frequency of this genetic alteration in our patients compared to controls and its relevance to the pathophysiology of inflammatory diseases stimulated the search of its presence in all 402 study subjects. Interestingly, the *IL1RL1* variant, absent in controls, was confirmed in more than 19% of FMF patients belonging to the different *MEFV* subgroups. The *IL1RL1* variant was found even higher in FMF patients carrying a single or no mutation in *MEFV* gene.

The *IL1RL1* gene product, which has been given the alias ST2, is defined as the IL‐33 receptor.^40^, ^41^ ST2 is a member of the IL‐1 receptor family. There are two main isoforms: a membrane‐bound form (ST2), which promotes NF‐κB signalling, and a soluble receptor (sST2) which prevents its signalling. ST2/IL‐33 pathway has been implicated in a wide range of disease settings, in anti‐inflammatory responses and homeostasis, and thus, signalling must be strictly regulated.^42^ Dysregulation of ST2/IL‐33 signalling and sST2 production have been implicated in a variety of inflammatory diseases.^43^, ^44^ ST2 contains an extracellular domain, which binds IL‐33, a transmembrane domain, and an intracellular domain called a Toll/interleukin 1 receptor (TIR) domain. The novel variant (2‐Kb deletion) of *IL1RL1* gene, reported in the present study, covers the totality of exon 11 encoding the TIR domain. Therefore, this deletion could lead to the disruption of the IL‐33/ST2 signalling.

Although this current study, showing the presence of many rare variants in genes associated with auto‐inflammatory disorders and a novel variant (a 2.1‐Kb deletion) in exon 11 of *IL1RL1* gene (NM_016232) in atypical FMF patients (carrying a single or no mutation in *MEFV*), supports the multigenic inheritance model of FMF, a large‐scale typing in Lebanese FMF patients is needed. The small number of healthy control subjects included in the Genome sequencing analysis constitutes a potential limitation of our study. Replication of the present findings in other populations will be useful to determine whether the association between these genetic markers and FMF can be generalized. We believe that our findings could have potential implications in the diagnostic and disease management of FMF. The extreme variability of clinical presentation and disease severity of FMF constitute a significant challenge for clinicians. As pointed out by Gangemi et al,^45^ although the *MEFV* genotype‐phenotype correlation in FMF patients has been intensively investigated, a clear consensus has not yet been reached. Several hypotheses have been proposed to explain the clinical heterogeneity of FMF but the clinical and diagnostic dilemma remain unsolved. While the current study showed that FMF patients carried a large spectrum of variants in several inflammatory genes, certain variants seem to be quite prevalent in patients carrying a single or no mutation in *MEFV* gene including variants in the 4 genes (*PRF1*, *STXBP2, RAB27A* and *UNC13D*) associated with FHL and the novel variant that we have discovered in the *IL1RL1* gene. A more holistic approach integrating clinical data and comprehensive genetic investigations, not limited to *MEFV*, could constitute the most effective diagnostic process to confirm or refute the diagnosis of FMF. A large phenotype‐genotype study will be undertaken to identify potential associations between the numerous genetic variants herein reported and specific clinical features of FMF.

In conclusion, this study provides novel evidence supporting a multigenic model of inheritance in FMF. The novel *IL1RL1* gene variant that we have identified in a significant proportion of our patients qualifies as an additional genetic marker for FMF. These findings pave the way for future studies that would provide more insight into the molecular mechanisms underlying FMF and for the design of new and more effective genetic tests for the diagnosis of FMF.

## CONFLICTS OF INTEREST

The authors confirm that there are no conflicts of interest.

## AUTHOR CONTRIBUTION

**Meenakshi Umar:** Data curation (equal); Investigation (equal); Methodology (equal); Writing‐original draft (lead); Writing‐review & editing (supporting). **Andre Megarbane:** Conceptualization (supporting); Data curation (equal); Investigation (equal); Resources (lead). **Jingxuan Shan:** Investigation (equal); Methodology (supporting); Writing‐original draft (supporting). **Najeeb Syed:** Data curation (equal); Formal analysis (equal). **Eliane Chouery:** Data curation (equal); Formal analysis (equal). **Elbay Aliyev:** Data curation (equal); Formal analysis (equal). **Puthen Jithesh:** Data curation (supporting); Formal analysis (equal). **Ramzi Temanni:** Data curation (supporting); Formal analysis (equal). **Issam Mansour:** Data curation (supporting); Resources (supporting). **Lotfi Chouchane:** Conceptualization (equal); Project administration (supporting); Supervision (equal); Writing‐original draft (equal); Writing‐review & editing (equal). **Aouatef Ismail Chouchane:** Conceptualization (equal); Funding acquisition (lead); Project administration (lead); Supervision (lead); Writing‐original draft (equal); Writing‐review & editing (equal).

## Supporting information

Fig S1

Table S1

## Data Availability

All the variants reported here have been submitted to LOVD website (https://www.lovd.nl).
