# Supplementary material for: Genome sequencing unveils mutational landscape of the familial Mediterranean fever: Potential implications of IL33/ST2 signalling
Source: J Cell Mol Med. 2020 Aug 27;24(19):11294–306. doi: 10.1111/jcmm.15701 (PMC7576248; doi:10.1111/jcmm.15701)
Supplement: Supplementary file 1 — Fig S1 [file JCMM-24-11294-s001.docx]

**
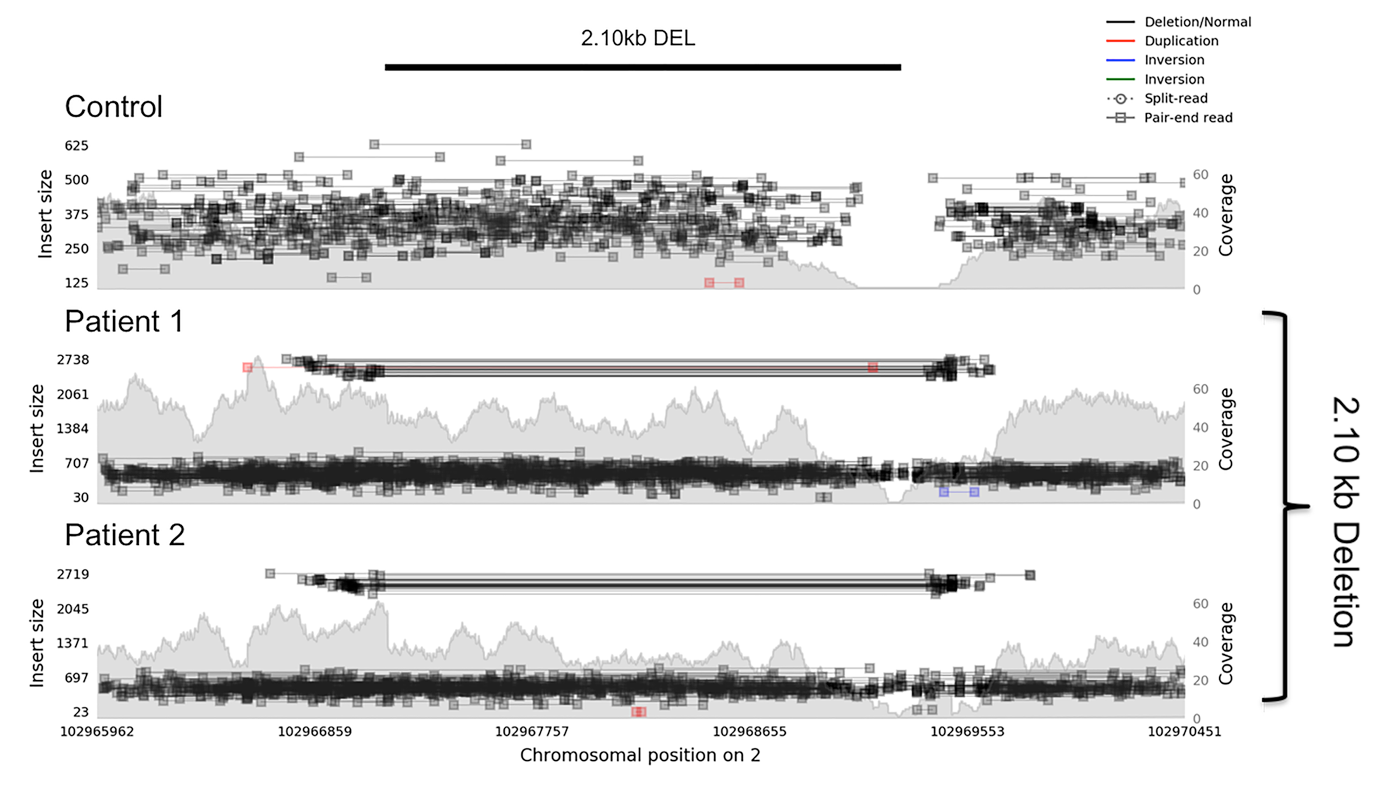
**

**Supplementary Figure 1:** A representative diagram of the novel variant (2.1 Kb deletion) of *IL1RL1* (NM_016232, NC_000002.11:g.102967165_102969288del), revealed by WGS and CNV analysis in FMF patients.
