# Supplementary material for: Genome sequencing unveils mutational landscape of the familial Mediterranean fever: Potential implications of IL33/ST2 signalling
Source: J Cell Mol Med. 2020 Aug 27;24(19):11294–306. doi: 10.1111/jcmm.15701 (PMC7576248; doi:10.1111/jcmm.15701)
Supplement: Supplementary file 2 — Table S1 [file JCMM-24-11294-s002.docx]

**Supplementary Table 1**: Summary of variants in *MEFV* gene, auto-inflammatory disorders (AID) associated genes and inflammatory genes per FMF patients.

| **Patient ID** | **No. of Exonic MEFV variants** | ***MEFV* variants** | **AID associated variants** | **Inflammatory genes variants** | **Structural variant** |
| --- | --- | --- | --- | --- | --- |
| FMF1 | 2 | Val726Ala (Het), Met694Val (Het) |  |  |  |
| FMF2 | 2 | Met694Val (Homo) | *PRF1* Ala91Val (Het),  *STXBP2* p.Thr345Met (Het), *NOD2* p.Arg227fs*145 (Het) |  |  |
| FMF3 | 2 | Val726Ala (Homo) |  |  |  |
| FMF4 | 2 | Val726Ala (Homo) | *UNC13D p.Met204Val* (Het) | *IFNAR2* p.Thr204Arg (Het) |  |
| FMF5 | 2 | Val726Ala (Het), Glu148Gln (Het) |  | *IFNAR2* p.Thr204Arg (Het) |  |
| FMF6 | 2 | Val726Ala (Het), Met694Val (Het) |  |  |  |
| FMF7 | 2 | Val726Ala (Het), Met694Val (Het) |  | *IFNAR2* p.Thr204Arg (Het) |  |
| FMF8 | 2 | Met694Ile (Het), Met680Ile (Het) |  | *IFNAR2* p.Thr204Arg (Het) |  |
| FMF9 | 2 | Arg408Gln (Het), Pro369Ser (Het), Glu148QGln (Het) |  |  |  |
| FMF10 | 2 | Val726Ala (Homo) | *IFIH1* p.Glu376Lys (Het), *CARD14* p.Arg80Gln (Het) |  |  |
| FMF11 | 2 | Val726Ala (Het), Met680Ile (Het) | *PRF1* Arg385Trp (Het) | *IL1R2* p.Ile311Thr (Het) |  |
| FMF12 | 1 | Met694Val (Het), c.-397C>G (Het) | *PSTPIP1* c.37-10081C>G (Het) | *ERAP2* p.Thr347Met (Het) |  |
| FMF13 | 1 | Met694Val (Het), c.-1309G>A (Het) | *PLCG2* p.Met28Leu (Het) | *TNFRSF8* p.Arg504Gln (Het) | *IL1RL1* del (Het) |
| FMF14 | 1 | Met694Ile (Het) | *PRF1* p.Ala91Val (Het), *TNFRSF11A* p.Arg450Trp (Het) | *IFNAR2* p.Thr204Arg (Het) | *IL1RL1* del (Het) |
| FMF15 | 1 | Met694Val (Het) |  | *CASP14* p.Gly140Ser (Het) | *IL1RL1* del (Het) |
| FMF16 | 1 | Ala744Ser (Het) |  | *IL12A* p.Val211Met (Het) | *IL1RL1* del (Het) |
| FMF17 | 1 | Lys695Arg (Het) |  | *IL1R2* p.Ile311Thr (Het) |  |
| FMF18 | 1 | Met694Val (Het) | *PRF1* p.Arg385Trp (Het), *RAB27A* p.Tyr6Cys (Het); *NOD2* p.Arg744Trp (Het) | *TNFRSF4* p.Cys128* (Het) |  |
| FMF19 | 1 | Met694Val (Het) | *UNC13D* p.His224Tyr (Het) | *TRAFD1* p.Glu303Ala (Het) |  |
| FMF20 | 1 | Arg408Gln (Het), Pro369Ser (Het) |  | *IFNAR1* p.Trp318Cys (Het) | *IL1RL1* del (Het) |
| FMF21 | 1 | Met694Ile (Het) |  | *IFNAR1* p.Trp318Cys (Het) | *IL1RL1* del (Het) |
| FMF22 | 1 | Arg408Gln (Het), Pro369Ser (Het) |  |  |  |
| FMF23 | 1 | Met694Val (Het) | *PRF1* p.Ala91Val (Het) | *RAB27B* p.Ala92Thr (Het) | *IL1RL1* del (Het) |
| FMF24 | 1 | Met694Val (Het) | *TNFAIP3* p.Arg136Cys (Het) |  |  |
| FMF25 | 1 | Met694Val (Het) | *NLRP3* p.Thr954Met (Het), *SH3BP2* p.Arg534Trp(Het) | *IL1R2* p.Ile311Thr (Het) |  |
| FMF26 | 1 | Lys695Arg (Het) |  |  |  |
| FMF27 | 1 | Val726Ala (Het) |  |  |  |
| FMF28 | 1 | Val726Ala (Het) | *STXBP2* p.Thr345Met (Het), *TNFRSF11A* p.Asp412Tyr(Het) | *IFNAR2* p.Thr204Arg (Het) | *IL1RL1* del (Het) |
| FMF29 | 1 | Arg408Gln (Het), Pro369Ser (Het) |  |  | *IL1RL1* del (Het) |
| FMF30 | 1 | Ala744Ser (Het) | *AP1S3* p.Phe4Cys (Het) |  |  |
| FMF31 | 0 | c.-123A>G (Het) |  |  |  |
| FMF32 | 0 |  | *NOD2* p.Trp709*(Het) | *IL17RB* p.Gly177Arg (Het),  *LILRB1* p.Gly333Cys (Het) |  |
| FMF33 | 0 |  | *PSTPIP1* p.Thr68Lys (Het) | *IFNAR2* p.Thr204Arg (Het), *IL1A* p.Asp176His (Het) |  |
| FMF34 | 0 |  |  | *NLRP2* p.Gly891Val (Het) |  |
| FMF35 | 0 |  | *STXBP2* p.Thr345Met (Het) |  |  |
| FMF36 | 0 |  |  | *ERAP1* p.Gly460Arg (Het) |  |
| FMF37 | 0 |  |  | *TNFRSF4* p.Cys128*(Het) |  |
| FMF38 | 0 |  |  | *ERAP2* p.Thr347Met (Het) |  |
| FMF39 | 0 |  |  |  |  |
| FMF40 | 0 |  |  | *TNFSF9* p.Arg239Gln (Het), *IL17RB* p.Gly177Arg (Het), *NLRC3* p.Ala801Thr (Het), *ICAM1* p.Arg367Cys (Het) |  |
| FMF41 | 0 |  |  | *TLR1* p.Met338Thr (Het), *ERAP2* p.Thr347Met (Het) |  |
| FMF42 | 0 |  |  |  |  |
| FMF43 | 0 |  |  |  |  |
| FMF44 | 0 |  | *SH3BP2* p.R505W (Het) | *IL20* c.225+1G>T (Het) |  |
| FMF45 | 0 |  | *CARD14* p.R597W (Het) |  |  |
| FMF46 | 0 |  |  |  |  |
| FMF47 | 0 |  | *NOD2* c.2883-2A>G (Het) |  |  |
| FMF48 | 0 |  | *IFIH1* p.P866L (Het) | *IL17RB* p.Gly177Arg (Het) |  |
| FMF49 | 0 |  |  | *IL17RD* p.Pro566Ser (Het), *ERAP2* p.Thr347Met (Het), IFNB1 p.Ile166Met(Het) |  |
| FMF50 | 0 |  | *PRF1* p.R385W (Het) | *NLRX1* p.Val494M (Het) |  |
